# Preparation and Performance of Porous Carbon Nanocomposite from Renewable Phenolic Resin and Halloysite Nanotube

**DOI:** 10.3390/nano10091703

**Published:** 2020-08-29

**Authors:** Xiaomeng Yang, Xiaorui Zeng, Guihong Han, Dong Sui, Xiangyu Song, Yongsheng Zhang

**Affiliations:** 1 School of Chemical Engineering, Zhengzhou University, Zhengzhou 450001, China; yangxiaomeng@126.com (X.Y.); xrzeng@126.com (X.Z.); guihong-han@hotmail.com (G.H.); 2 Key Laboratory of Function-Oriented Porous Materials, College of Chemistry and Chemical Engineering, Luoyang Normal University, Luoyang 471934, China; suidonghy@mail.nankai.edu.cn

**Keywords:** halloysite nanotube, phenol-furfural resin, composite porous carbon, supercapacitors

## Abstract

The growing demand for high performance from supercapacitors has inspired the development of porous nanocomposites using renewable and naturally available materials. In this work, a formaldehyde-free phenolic resin using monosaccharide-based furfural was synthesized to act as the carbon precursor. One dimensional halloysite nanotube (HNT) with high porosity and excellent cation/anion exchange capacity was mixed with the phenol-furfural resin to fabricate carbonaceous nanocomposite HNT/C. Their structure and porosity were characterized. The effects of the halloysite nanotube amount and carbonization temperature on the electrochemical properties of HNT/C were explored. HNT/C exhibited rich porosity, involving a large specific surface area 253 m^2^·g^−1^ with a total pore volume of 0.27 cm^3^·g^−1^. The electrochemical performance of HNT/C was characterized in the three-electrode system and showed enhanced specific capacitance of 146 F·g^−1^ at 0.2 A g^−1^ (68 F·g^−1^ for pristine carbon) in electrolyte (6 mol·L^−1^ KOH) and a good rate capability of 62% at 3 A g^−1^. It also displayed excellent cycle performance with capacitance retention of 98.5% after 500 cycles. The symmetric supercapacitors with HNT/C-1:1.5-800 electrodes were fabricated, exhibiting a high energy density of 20.28 Wh·Kg^−1^ at a power density of 100 W·Kg^−1^ in 1 M Na_2_SO_4_ electrolyte. The present work provides a feasible method for preparing composite electrode materials with a porous structure from renewable phenol-furfural resin and HNT. The excellent supercapacitance highlights the potential applications of HNT/C in energy storage.

## 1. Introduction

Electric double-layer capacitors (EDLCs), known as one of the typical supercapacitors, accumulate positive and negative charges at the interface of electrode material and electrolyte [1,2]. EDLCs have been utilized in hybrid electric vehicles, portable electronic devices and electric toys due to their excellent performance in power density (>10 kW·Kg^−1^), charge–discharge processes, cycle life and safety [3,4]. However, EDLCs suffer from low energy density and poor rate performance due to the low electrical conductivity and the pore volume of the materials used [5]. To elevate the storage capacity of EDLCs, carbon materials such as activated carbon [6], carbon nanotubes [7], graphene [8], carbon nanofibers [9] and carbide-derived carbons have been used as electrodes, because of their high specific surface area, porosity and low capital cost [10,11]. Therefore, conducting materials with a high surface-area, especially an electrochemically accessible surface area, are highly desired to elevate the storage capacity of EDLCs [12,13].

Among the potential alternatives, porous carbons from biobased phenolic resins have attracted enormous attention due to their well-controlled structure, high specific surface area and pore volume, as well as efficient performance in supercapacitors. Phenolic resin is typically prepared by polycondensation between phenol and formaldehyde in an acidic or alkali medium [14,15]. It has been widely applied from molding compounds to electrode materials [16,17]. Lei et al. [18] obtained activated carbon (AC) from phenolic resin after physical activation treatment, which exhibited a surface area of up to 1600 m^2^·g^−1^ and a specific capacitance of 160 F·g^−1^ at 1 mA·cm^−2^. Chen et al. [10] obtained rich nitrogen-doped ordered mesoporous carbon (N-OMC) with phenolic resin as a carbon precursor, achieving a specific surface area of 320 m^2^·g^−1^ (553 m^2^·g^−1^ for OMC) and a specific capacitance of 216 F·g^−1^ at 0.1 A g^−1^ (127 F·g^−1^ for OMC). The high physical and electrochemical properties suggested that phenolic resin is a good carbon source for EDLCs. However, the increasing concern is the carcinogenic formaldehyde that threatens human health and environmental friendliness [19]. Many researchers have devoted their efforts to innovate synthetic methods, in particular to seeking less toxic, green alternatives to formaldehyde. Zhang et al. [20] developed formaldehyde-free phenolic resin by using sugar-derived hydroxymethylfurfural (HMF) and furfural to substitute formaldehyde. This work inspired us to shift the focus from traditional phenolic resins to environmentally friendly biobased resins and to replace carbon materials from petrochemical products. Furthermore, the specific capacitance and the rate performance of the phenolic resin-based carbon materials are comparatively low [10,21]. The most effective strategy for enhancing the capacitive performance of phenolic resin-based porous carbon materials is to fabricate hierarchical nanocomposites.

In recent years, nanostructured materials with a high specific surface area and porosity have attracted great attention by providing proper contact with the electrolyte. Halloysite nanotube (HNT) is a natural mineral with a hollow channel and high specific surface area [21]. HNT, 1D tubular mineral with Al: Si ratio of 1:1, has aroused considerable attention [22,23]. Its unique structure can provide channels for electrolyte ions to cut down the transmission distance and enhance the specific capacitance. Huang et al. [24] prepared nanocomposites with HNT and polyaniline, obtaining specific capacitance of 137 F·g^−1^ at 0.5 A·g^−1^. Furthermore, the abundant hydroxyl groups on its surface and the excellent cation/anion exchange capacity facilitate the transmission rate of OH^-^ groups, thus the rate capability and the wettability of carbon materials were promoted [25,26,27,28]. The large surface area and high porosity make HNT a promising composite component of phenol-furfural resin [29].

Herein, we propose a robust strategy to fabricate an HNT/C nanocomposite with high specific surface area, and micro/mesopores by using HNT and phenol-furfural resin as precursor. This work aims to explore the optimal composite ratio and carbonization temperature. The electrochemical performances of the composite are expected to improve through the synergistic effect of the two components. The main challenges are how to make better use of the framework and functional groups of HNT, and offset the poor conductivity of HNT by carbon materials. Among them, we evaluated the possibility of using HNT/C nanocomposite to fabricate electrodes for EDLC. Electrochemical evaluations showed that the as-prepared HNT/C exhibited considerably improved capacitive performance compared with pristine carbon and HNT.

## 2. Experimental

### 2.1. Materials

Phenol (C_6_H_6_O) (A.R.), furfural (C_5_H_4_O_2_) (A.R., 99%), potassium carbonate (K_2_CO_3_) (A.R., 99%) and potassium hydroxide (KOH) (A.R., 90%) were supplied by Aladdin Reagent Co. Ltd., Shanghai, China. Sodium sulfate (Na_2_SO_4_) (A.R., 99%), ethanol absolute (C_2_H_6_O) (A.R., 99.7%) and polyvinylidene fluoride (PVDF) (M_w_ ~ 400,000) were purchased from Macklin Reagent Co. Ltd., Shanghai, China. Hydrochloric acid (A.R.) was supplied by Luoyang Haohua Chemical Reagent Co. Ltd., Luoyang, China. Halloysite nanotube (HNT) was supplied by Runwo Material Technology Co. Ltd., Guangzhou, China. The conductive acetylene black was supplied by YiboRui Chemical Co. Ltd., Tianjin, China. All the chemicals were used without further purification.

### 2.2. Materials Preparation

#### 2.2.1. Synthesis of Renewable Phenolic Resin

Typically, 9.41 g phenol and 0.56 g K_2_CO_3_ as a basic catalyst were mixed homogeneously in a 250 mL three-neck flask with condenser. The obtained mixture was then transferred into the oil bath where the flask was heated to 70 °C. Subsequently, 8.07 g of furfural was added dropwise and polymerized for 1.5 h. Then the temperature was elevated to 120 °C, 3.46 g of the remaining furfural was charged slowly into the medium and further polymerized for 1.5 h. The biobased phenolic resin was purified by removing volatile matters using a rotary evaporator and vacuum oven at 55 °C.

#### 2.2.2. Pretreatment of HNT

In order to remove impurities, 1 g of halloysite nanotube was pretreated with 20 mL hydrochloric acid (5 M) at 65 °C with continuous agitation for 6 h. It was further washed with deionized water till pH 7 and was dried at 60 °C for 8 h. Finally, it was calcined at 550 °C for 5 h in a muffle furnace.

#### 2.2.3. Preparation of HNT/C

The phenolic resins were diluted with anhydrous ethanol to form 50 wt% solid content solution. The HNT and phenolic resin solution (50 wt%) were homogeneously mixed in mass ratios of 1:1.5, 1:2.0 or 1:2.5. The obtained mixture was then sealed with consecutive agitation for 6 h at 65 °C to form a clad on the surface of HNT. The product was refined by rotary evaporator and dried in a vacuum oven at 65 °C for 12 h. Then, the resin precursor was cured following the program of 120 °C/1 h, 150 °C/1 h, and 180 °C /1 h. Finally, they were carbonized in a tubular furnace at 700, 800 or 900 °C for 2 h under N_2_ flow. The obtained materials were denoted as HNT/C-X-Y, where X and Y represented of the mass ratio of HNT to phenol-furfural resin and carbonization temperature, respectively.

### 2.3. Characterizations

The surface morphology of those samples were observed by scanning electron microscope (SEM, SU 3500) manufactured by Hitachi, Tokyo, Japan.

X-ray diffraction (XRD) patterns were obtained on a D8 Advance diffractometer (Bruker, Karlsruhe, Germany) using Cu Kα radiation (λ = 0.1514178 nm).

Raman analysis was performed using LabRAM HR Evo from Paris, France, and the laser excitation was 532 nm.

Fourier transform infrared (FT-IR, PerkinElmer, Massachusetts, USA) spectra were obtained between the wavenumbers of 500 to 4000 cm^−1^.

The chemical binding state of the surface was characterized by X-ray photoelectron spectroscopy (XPS) on an AXIS Supra spectrometer, Shimadzu, Tokyo, Japan.

Brunaner-Emmett-Teller (BET) analysis was conducted by using ASAP 2020 system (Micrometitics, GA, USA) to determine specific surface area (*S_BET_*), total pore volume (*V_T_*) and pore size distribution. The carbon materials were degassed at 300 °C for 3 h before analysis.

### 2.4. Electrochemical Measurements

The HNT/C nanocomposite materials, carbon black and polyvinylidene fluoride (PVDF) were uniformly mixed and grounded in the ratio of 8:1:1 by weight. A portion of 3–5 mg of the mixture was coated on 1 × 2 cm^2^ nickel foam within a coating area of 1 × 1 cm^2^, and dried in reduced pressure at 55 °C overnight. Then the electrode material was pressed into 1 × 1 cm^2^ sheet under 10 MPa pressure. The three-electrode test was carried out in 6 M KOH solution, using a 1 × 1 cm^2^ Pt plate as the counter electrode, Hg/HgO as the reference electrode, and the HNT/C-X-Y nanocomposite as the work electrode, respectively. Two electrode sheets with the same mass were assembled into a symmetric supercapacitor, also known as a two-electrode test system, and aqueous Na_2_SO_4_ (1 M) was used as the electrolyte. The cyclic voltammetry (CV) and electrochemical impedance spectroscopy (EIS, 0.01 to 10^5^ Hz) were measured on electrochemical work stations (AUT86544, MetrohmCo. Ltd., Herishaw, Switzerland). Galvanostatic charge−discharge (GCD) properties were obtained using a battery test system (Land CT2001A, Wuhan Jinnuo Electronic Co. Ltd., Wuhan, China). The CV test was accomplished in the three-electrode test system range from −1 to 0 V, and the voltage range of the GCD test in the two-electrode test system was 0–1 V.

From the GCD and CV profile in the three-electrode system, the mass ratio capacitance of the electrode materials could be calculated according to Equations (1) and (2) respectively:

(1) $$C=\frac{I*\Delta T}{m*\Delta U}$$

(2) $$C=\frac{\int IdU}{2sm\Delta U}$$

where *C* (F·g^−1^), *I* (A), Δ*T* (s), Δ*U* (V), *s* (mV/s) and *m* (g) represent the mass ratio capacitance, the constant discharging current, the discharging time, the potential window, scanning rate and the weight of the total mass of electro material of a single electrode, respectively.

From the discharge curve in the two-electrode system, the specific energy density *E* and power density *P* can be obtained according to Equations (3)–(5) respectively:

(3) $$C=\frac{2I*\Delta T}{m*\Delta U}$$

(4) $$E=\frac{1}{2}C\Delta U^{2}$$

(5) $$P=\frac{E}{\Delta T}$$

where *E* (W·h kg^−1^) is the specific energy density and *P* (W·kg^−1^) is the specific power density. 

## 3. Results and Discussion

**Figure 1:**
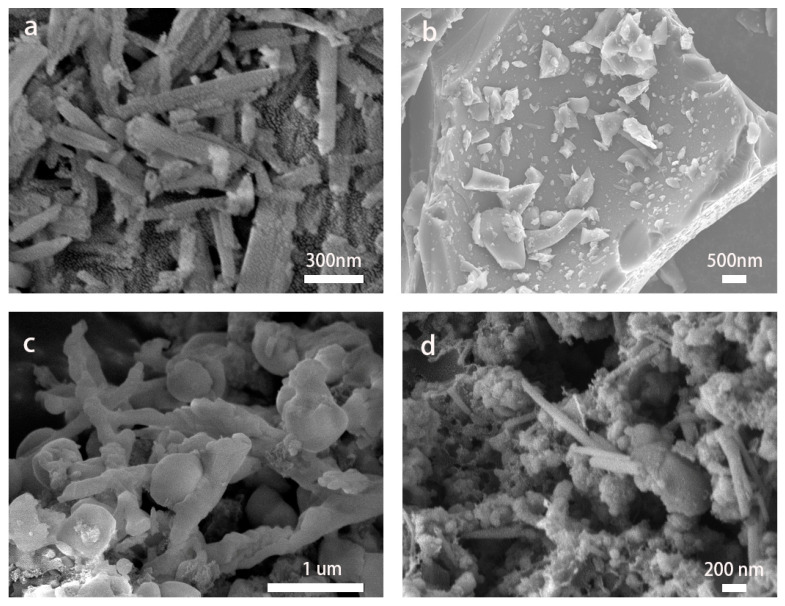
(**a**) SEM images of halloysite nanotube (HNT), (**b**) carbon derived from phenol furfural resin and (**c**,**d**) HNT/C-1:1.5-800 composite material.

The microstructure and morphology of HNT/C nanocomposites were firstly investigated by SEM images. As observed from Figure 1a, pristine HNT exhibits a typical hollow and thin-wall structure. The average outer diameter of HNT is approximately 100 nm with a uniform length. In addition, the unique hollow structure and appropriate size of HNT were vital to adjust the size of carbon-coated materials and improve the specific surface area. Therefore, HNT is believed to substantially improve the specific capacitance of the nanocomposite. As shown in Figure 1b, the conductive carbon material has a blocky microstructure without obvious pores. Therefore, HNT was introduced to enhance the electrochemical energy storage of pristine carbon derived from phenol furfural resin. Figure 1c,d exhibits the morphology of the HNT/C-1:1.5-800, which retained a tubular structure while the diameter of the nanocomposite increased to ~200 nm because of the coverage of phenol-furfural resin-derived carbon. Furthermore, the mass ratio of HNT to phenol-furfural resin showed a strong effect on the HNT/C diameter. Figure 1 confirms that the amorphous phenol-furfural resin-derived carbon shells with different thickness could be continuously wrapped on the HNT, demonstrating the strong shape and structure-directing role of HNT. Additionally, an interconnection network with pores constructed by considerable carbon microspheres could be clearly observed [18,30]. Meanwhile, the specific structure of the HNT/C may provide accessible electroactive sites and shorten ion/electron transport pathways.

**Figure 2:**
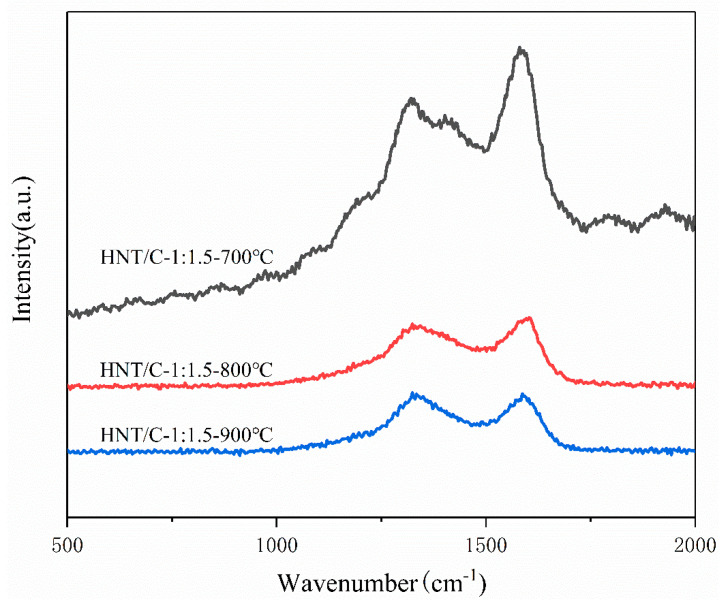
Raman spectra of HNT/C at different ratios and carbonization temperatures.

As demonstrated by XRD patterns of HNT/C samples (Figure S1), the PF polymer coated on the HNT was converted into an amorphous carbon shell upon the carbonization process. The broad diffraction peak centered at about 22.8° was assigned to the amorphous structure of carbon. However, compared with the sample of HNT/C-X-700, the diffraction width became slightly broader and the peak intensity slightly decreased for HNT/C-1:1.5-800 and HNT/C-1:1.5-900 samples, indicating the slight reduction of the ordered structures while increasing the carbonization temperature [10]. Meanwhile, after the carbonization process, remarkable micropores were created due to the decomposition and release of organic compounds. Increasing the temperature of carbonization caused a shift in the diffraction peaks of HNT/C-1:1.5-800 to the lower angle. This indicates that the unit cell parameters increased because the carbon structure expanded during high temperature carbonization [31,32,33]. As a result, the electrochemical performance was believed to be enhanced accordingly. Raman analysis was applied to further characterize the structure of the composite (Figure 2). All samples at different carbonization temperatures have two distinct peaks. The peaks at ~1350 cm^−1^ and ~1580 cm^−1^ are D and G bands, respectively [17]. The integral ratio (*I_G_*/*I_D_*) represents the graphitization degree of the material. The *I_G_*/*I_D_* values of the three samples at different temperatures were 3.03, 0.80 and 0.59, respectively. This indicates that the graphitization degree of the material decreases with the increase of carbonization temperature. This result is consistent with the XRD results.

**Figure 3:**
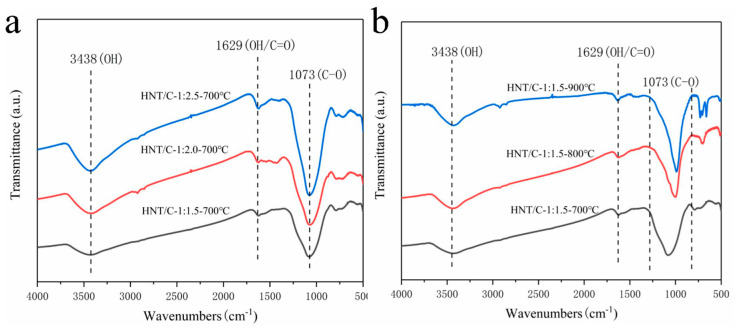
FT-IR spectra of (**a**) HNT/C-X-700 and (**b**) HNT/C-1:1.5-Y.

The FT-IR spectra of the HNT/C nanocomposites with different ratios and carbonization temperatures are given in Figure 3. The obvious absorption bands between 3300 and 3600 cm^−1^ can be attributed to the stretching vibrations of O−H bonds of HNT. This is particularly interesting for EDLCs because the high amount of hydroxyl groups on the HNT support can improve the diffusion of aqueous alkali electrolyte ions within the electrode material, that can increase the accessibility of active sites and the electrochemical performance of the electrodes [34]. It has been reported that the abundant hydroxyl group on the surface and interlayer of electrodes caused excellent cation/anion exchange capacity [35]. The weak absorbance at 1629 cm^−1^ belong to the bending vibrations of C=O and O–H bonds. The absorption peak at 1073 cm^−1^ corresponds to the ether groups which may be derived from furfural. Compared with pristine carbon materials, these oxygenated functional groups of HNT/C were expected to enhance hydrophilic surface property [34].

**Figure 4:**
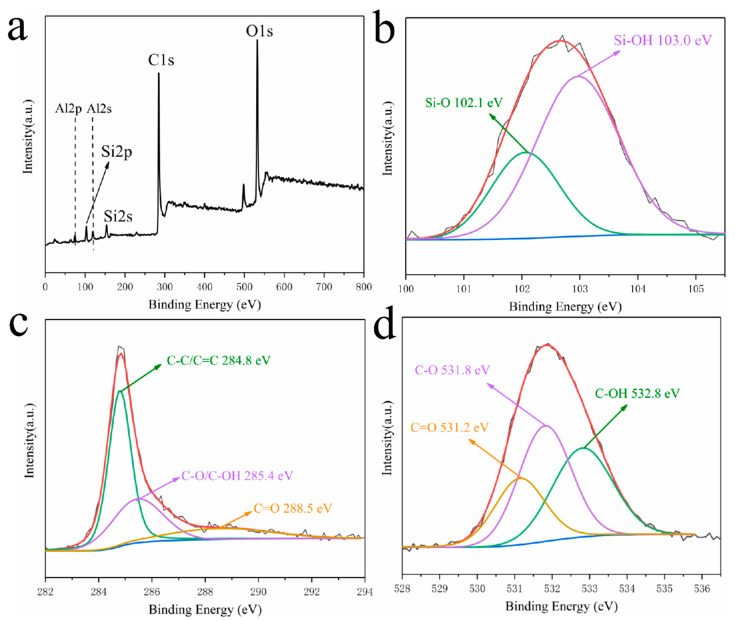
(**a**) Survey scan, deconvoluted XPS of (**b**) Si 2p, (**c**) C 1s, and (**d**) O 1s of HNT/C-1:1.5-800.

The XPS spectra of HNT/C-1:1.5-800 (Figure 4a) reveal the characteristic peaks of Al 2p (74.8 eV), Si 2p (102.1 eV), Al 2s (118.8 eV), Si 2s (153.8 eV), C 1s (284.8 eV), and O 1s (531.9 eV). There are some weak Al 2p, Si 2p, Al 2s and Si 2s peaks in the profile of HNT/C-1:1.5-800 owing to the HNT, demonstrating that the HNT was successfully incorporated into the carbon framework. In the case of Si-species (Figure 4b), two peaks at 102.1 and 103.0 eV can be considered as the Si–O and Si–OH groups, respectively [36]. The high resolution C1s peak shows the fitting peaks of three different contributions in Figure 4c. In general, the peak at 284.8 eV corresponds to aromatic sp^2^ hybridized carbon. The shoulder peak at 285.4 eV can be designated as the carbon atoms in the C−O of the phenolic structure. Weak peaks between 285.4 and 288.5 eV imply the presence of carbonyl or amide groups [37]. The high-resolution O1s spectra of HNT/C-1:1.5-800 (Figure 4d) can be deconvoluted into three peaks at binding energies of 531.2 eV (C=O, carbonyl group), 531.8 eV (C–O, epoxy) and 532.8 eV (C–OH, hydroxyl groups), respectively [38,39]. These functional groups are consistent with the FTIR spectra (Figure 3).

**Figure 5:**
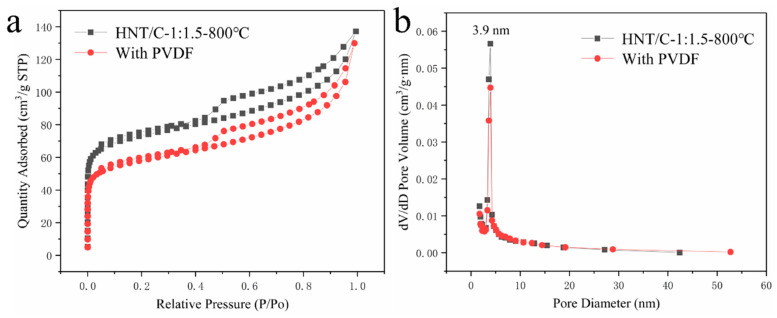
(**a**) Nitrogen adsorption/desorption isotherms and (**b**) pore size distributions (BJH) of HNT/C-1:1.5-800, with and without PVDF/carbon black.

Nitrogen adsorption-desorption isotherms were used to evaluate the surface area and pore size distribution of HNT/C-1:1.5-800 in Figure 5. Obviously, the sample shows a type IV curve with distinct type H3 hysteresis loop that confirms the micro/mesoporous structure. A remarkable rise at low pressure (*P*/*P*_0_ < 0.05) in the isotherms revealed the existence of micropores. Their increase in absorption capacity and hysteresis loop formation in the *P*/*P*_0_ range 0.1–0.8 also demonstrates capillary condensation within small-sized mesopores of HNT/C-1:1.5-800. Additionally, the adsorption of N_2_ at *P*/*P*_0_ substantially rose, approaching 1.0, implying large-sized mesopores or macropores in the sample. The pore size distribution obtained by the BJH (Barrett-Joyner-Halenda, test method for pore size distribution) method is shown in Figure 5b, indicating that HNT/C-1:1.5-800 has a narrow pore size distribution centered at 3.94 nm. Therefore, the HNT/C-1:1.5-800 has a pore structure which is mainly mesoporous and contains few micropores. Interconnected mesopores ensure that the electrolyte ions easily diffuse to the active sites of the surface by reducing transfer distance, thus improving their performance. We also analyzed the porosity of HNT/C-1:1.5-800 with binder (PVDF) and conductive agent (carbon black). The mixed material has an adsorption curve type and pore diameter distribution similar to that of HNT/C-1:1.5-800. Considering that the active component in the electrode material accounts for 80%, the theoretical BET surface area of the electrode material was 198 m^2^·g^−1^ [40,41]. As shown in Table 1, the *S_BET_* and *V_total_* of the material declined after mixing binder and conductive agent.

The detailed porosity parameters are also summarized in Table 1. When the carbonization temperature elevated to 800 °C, the specific surface area of the composite material increased to 248 m^2^·g^−1^. This indicates that the carbonization degree of the material is enhanced along with the rising temperature and forms a more porous structure. However, the specific surface area descends to 156 m^2^·g^−1^ and the pore diameter increases to 42.8 nm when the temperature rises further to 900 °C. This may be attributed to the collapse of narrow pores under higher carbonization temperature and large pores are formed. In addition, the specific surface area and total pore volume of the HNT/C-1:1.5-800 were 248 m^2^·g^−1^ and 0.20 cm^3^·g^−1^, respectively, which was almost twice that of HNT. This indicates that the unique structure of HNT can effectively improve the specific surface area of nanocomposites. As the mass ratio of HNT/C rose, the specific surface area gradually increased up to 254 m^2^·g^−1^. This illustrates that both the resin-derived carbon and HNT contribute to the specific surface area of the composite.

**Figure 6:**
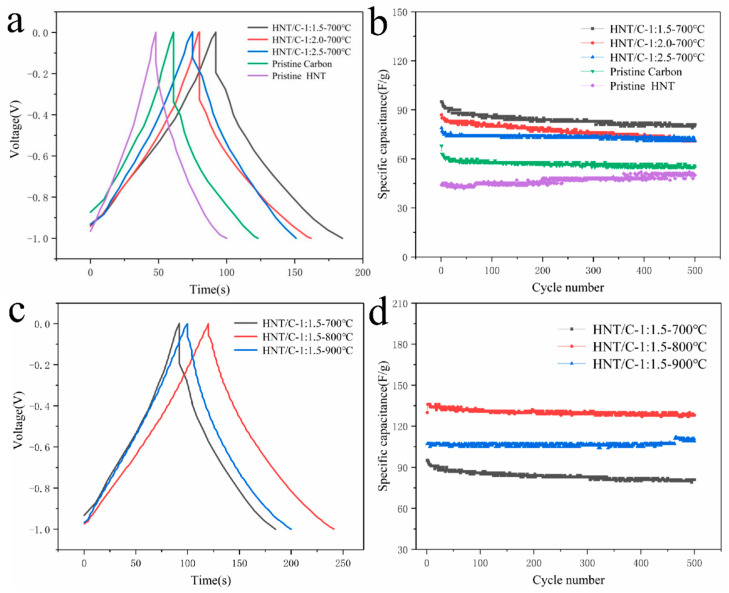
(**a**,**c**) Galvanostatic charge−discharge (GCD) curves and (**b**,**d**) cyclic performance curve of as-prepared samples.

The electrochemical properties of HNT/C samples were evaluated by GCD in aqueous KOH (6 M) with the voltage range from −1.0 to 0 V. The GCD measurements and cyclic performance curves of HNT/C-X-700, presented in Figure 6a,b, were used to investigate the effects of ratio of HNT. The charge–discharge curves of the specimens all showed triangular shapes with good symmetry, representing typical electrical double-layer capacitive behavior and excellent electrochemical reversibility. In addition, the discharge time was equivalent to the charging time (e.g., 121 versus 120 s for HNT/C-1:1.5-800 in Figure 6c), indicating excellent coulomb efficiency. Significant voltage drops were observed in GCD curves (Figure 6a), where pristine carbon presented the largest drop, corresponding with its higher internal resistance and a smaller specific capacitance. Figure 6b reveals similar phenomena, i.e., the specific capacitance of the composites was obviously greater than the pristine carbon or HNT, implying that the electrochemical properties of the composites have advanced. The incorporation of HNT could enhance the wettability and the ion migration rate of the HNT/C nanocomposite, and further promote the electrochemical capability as well as stability of active electrode materials. This can be ascribed to the synergistic effects of the unique structure of HNT and the good electrical conductivity of carbon. As the ratio of HNT to phenol-furfural resin increased from 1:1.5 to 1:2.5 in Figure 6a, the voltage drop of the sample increased and the specific capacitance decreased from 95 F·g^−1^ to 79 F·g^−1^. As the content of phenolic resin increases, the contribution of the HNT may diminish and the material would behave closer to carbon. From this point of view, the optimal ratio of HNT to phenol-furfural resin is set as 1:1.5.

As shown in Figure 6c, HNT/C-1:1.5-800 possessed a specific capacitance of 136 F·g^−1^ at 1 A g^−1^, which is superior to the HNT/C-1:1.5-700 (95 F·g^−1^) and the HNT/C-1:1.5-900 (107 F·g^−1^). With the raising of the carbonization temperature, the voltage drop of the materials was inhibited. This could be attributed to the higher carbonization degree and the more porous structure at a higher carbonization temperature. Figure S2 showed the GCD curves of HNT/C-1:1.5-800 in 6 M KOH electrolyte solution at varying current densities (0.2 to 3 A g^−1^). HNT/C-1:1.5-800 exhibited excellent specific capacitances of 146 and 90 F·g^−1^ at 0.2 and 3 A·g^−1^, respectively. The quasi-triangular with good symmetry could be seen in the GCD curves of HNT/C-1:1.5-800 even at a high current density of 3 A g^−1^, which indicated an excellent rate performance of the HNT/C-1:1.5-800. In order to evaluate the effects of carbonization temperature on the cycle stability, the cycle life tests were conducted at 1 A·g^−1^ (Figure 6d). For HNT/C-1:1.5-800 electrodes, 98.5% of the initial specific capacitance was retained after 500 cycles. The HNT/C-1:1.5-800 showed a much better performance than the other samples at 700 and 900 °C, which was attributed to the large specific surface area and proper porosity.

To analyze the self-discharge effect of HNT/C-1:1.5-800 as the electrode, it was charged to 0.34 V by constant current and the variation of voltage over time was recorded in Figure S3. After 30 min, the voltage descended to 0.17 V and finally stabilized at −0.29 V. The self-discharge effect is commonly recognized [42]. However, HNT/C-1:1.5-800 has a faster discharge rate, which is mainly due to impurities and charge leakage in the material [42]. Therefore, it is necessary to prepare electrode materials with large surface area and rich pore structure to release the self-discharge effect which remains challenging in the commercial application of supercapacitors.

**Figure 7:**
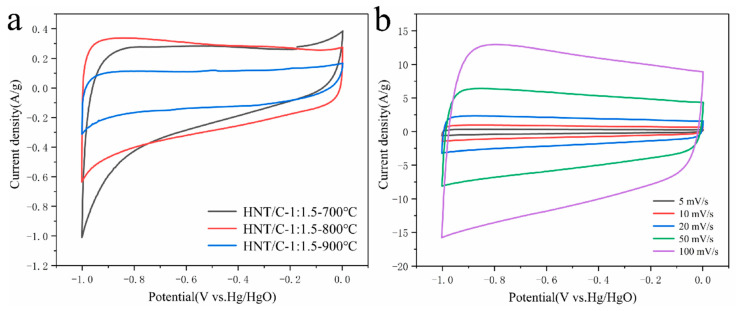
(**a**) The cyclic voltammetry (CV) curve of HNT/C-1:1.5-Y at scan rates of 5 mV/s. (**b**) Cyclic voltammograms of the HNT/C-1:1.5-800 electrode materials at scan rates of 5, 10, 20, 50, and 100 mV/s.

The electrochemical performance was also characterized by CV analysis. As shown in Figure 7a, HNT/C-1:1.5-Y samples with different carbonization temperature were performed at scan rates of 5 mV/s. A redox peak was not observed in the CV curve, demonstrating the formation of an efficient electric double layer [43]. As was expected, the largest CV curve integral area of HNT/C-1:1.5-800 is in line with the highest specific capacitance, which is consistent with the GCD measurement (Figure 6c,d). According to formula *C* = *I*/(*S_BET_* * *s*) [44], where *I* (A) and *s* (mV/s) represent the current and scanning rate, respectively, the electric double-layer capacitance was investigated in the form of cyclic voltammetry (Figure S5). At scan rates of 5 mV/s, the EDL capacity of HNT/C-1:1.5-800 achieved 0.5 µF·g^−1^, which was substantially higher than that of HNT/C-1:1.5-700 (0.47 µF·g^−1^) and HNT/C-1:1.5-900 (0.28 µF·g^−1^). This indicates that the pore structure of HNT/C-1:1.5-800 is close to the size of electrolyte ions, promoting ion transfer during charging/discharging processes. HNT/C-1:1.5-900 showed a flat CV curve, implying that the pore diameter was not suitable for ion adsorption, while the BET result also gave the same result [44]. This implies that HNT/C-1:1.5-800 with proper porosity could contribute a large electrochemical surface area, which is consistent with the abovementioned electrochemical test results.

Figure 7b shows typical CV curves of the HNT/C-1:1.5-800 at various scan rates ranging from 5 to 100 mV/s. According to the calculation of the integral area of the CV curve, the specific capacitance rose from 117 to 209 F·g^−1^ with the increase of scanning rate. Obviously, the CV curve remained approximately rectangular shape even at a high scan rate of 100 mV/s, demonstrating the excellent charge propagation and rate capability [13]. Furthermore, the specific capacitance contribution of HNT/C-1:1.5-800 at a scanning rate of 5 mV/s was calculated based on Trasatti’s Method [45,46]. According to formula *C* = 4.852 * *V*^−0.5^ + *C_EDL_*, V is the scan rate, the proportion of *C_EDL_* accounted for 41%.

**Figure 8:**
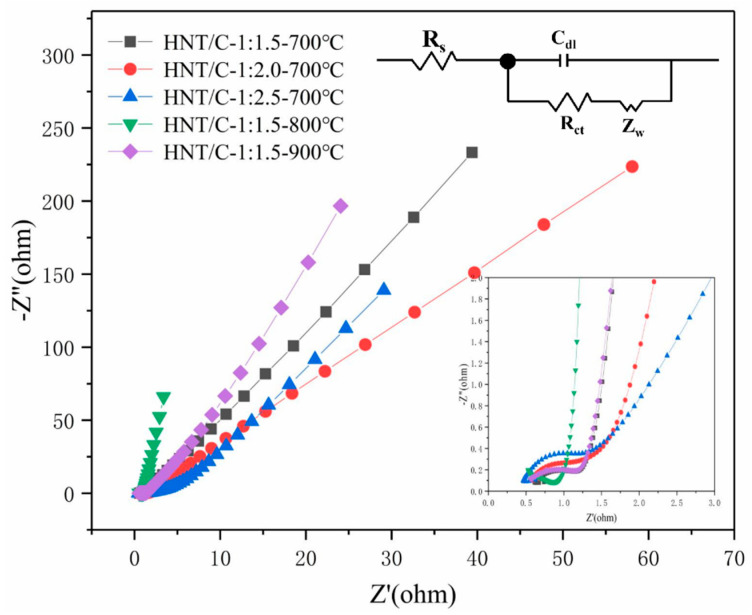
Nyquist impedance plot of HNT/C composite material. The insets are the equivalent electrical circuit diagram and enlarged high-frequency region of the plots.

To further understand the capacitive behavior of HNT/C, EIS analysis was performed (see Figure 8). The linear relationship in the low frequency region implies the diffusion resistance and ideal capacitive performance. A semicircle in the high frequency region corresponds to the charge transfer resistance of the HNT/C-X-Y [18,47]. All samples in the low frequency region showed good linear shape, representing typical EDLCs behavior. As observed from the inset of Figure 8, the slope decreases as the proportion increases, indicating that the diffusion resistance of the material enhances. HNT/C-1:1.5-700 presented the smallest semicircle diameter in the high frequency region, indicating its small resistance, which was consistent with previous electrochemical measurements. Compared with HNT/C-1:1.5-700, HNT/C-1:1.5-900 exhibited lower diffusion resistance. Although the charge transfer resistances of them were close in the high frequency region, the total resistance of HNT/C-1.1.5-900 was low, which was confirmed by the greater specific capacitance of HNT/C-1:1.5-900. The EIS data of HNT/C-1:1.5-900 was fitted using ZView software (Figure S5). Compared with the experimental data of EIS, the red fitted curve had a similar charge transfer resistance at high frequency, except for the deviation in low frequency regions. The frequency domain had a fitting match to within 17% while the high frequency region had a high fitting degree [48]. This model represents the basic dynamic behavior of the electrochemical system in this work. Apparently, the HNT/C-1:1.5-800 possessed the steepest slope of the curves and the minimum diameter of semicircle, suggesting the lowest charge transfer resistance and ion diffusion resistance [49]. The shortest length of HNT/C-1:1.5-800 in the vertical region indicated its largest specific capacitance, which corresponded with the results of GCD and CV [10,18]. Table 2 summarizes the electrochemical properties of the different materials, based on phenolic resin or HNTs reported by other researchers. Obviously, our prepared porous HNT/C composites exhibited superior electrochemical performance to other materials.

**Figure 9:**
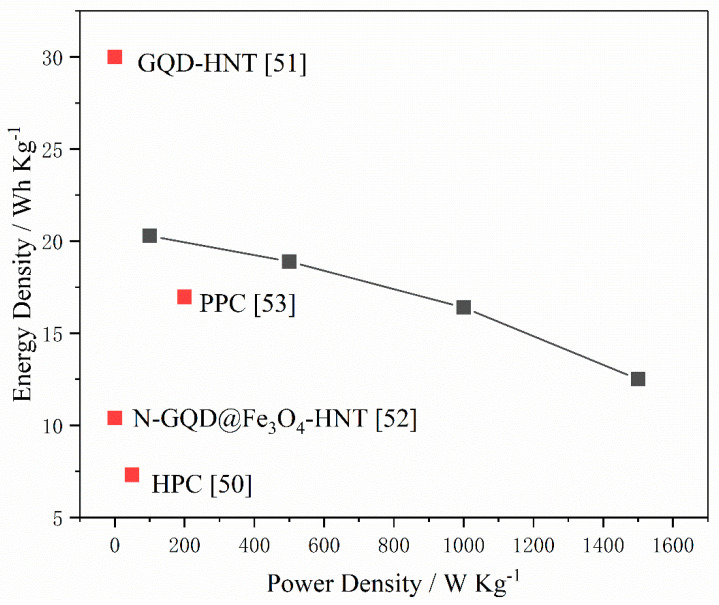
The Ragone plots of the symmetric cell and comparative performance of the symmetric cell versus previously reported ones (References: N-GQD@Fe_3_O_4_-HNT [52], HPC [50], GQD-HNT [51], PPC [53]).

Symmetric supercapacitors were assembled using two identical HNT/C-1:1.5-800 electrodes in aqueous Na_2_SO_4_ electrolyte (1 M) to investigate electrochemical cell performance. The HNT/C-1:1.5-800 symmetric supercapacitor could supply a maximal energy density of 20.28 Wh·Kg^−1^ where the power density was 100 W·Kg^−1^ (Figure 9). Its performance was better than the previously reported symmetrical supercapacitor based on phenolic resin or HNT, such as N-doped graphene quantum dots@Fe_3_O_4_-halloysite nanotubes [52], graphene quantum dots-halloysite nanotube [51], hierarchical porous carbons [50] and P-doped porous carbon [53]. The excellent power and energy density of HNT/C-1:1.5-800 may be ascribed to the large surface area and the hierarchical pore structures. The performance of symmetric supercapacitors of other samples is shown in Figure S6. The results also reveal that HNT/C-1:1.5-800 has the highest energy density at the same power density. Accordingly, HNT/C-1:1.5-800 demonstrates potential as an excellent electrode material for energy storage.

## 4. Conclusions

In summary, the HNT/C nanocomposites were prepared by using HNT and formaldehyde-free phenol-furfural resin synthesized from phenol and sugar-based furfural. The main challenges are how to make better use of the framework and functional groups of HNT, and offset the poor conductivity of HNT with carbon materials. In particular, the HNT/C had a high specific area (254 m^2^·g^−1^) with a total pore volume 0.27 cm^3^·g^−1^, which shortened the ion transport distance, increased the contact area between the material and electrolyte and exposed more active sites. Therefore, the HNT/C-1:1.5-800 delivered a high specific capacitance of 146 F·g^−1^ at 0.2 A·g^−1^ and outstanding rate performance (62% capacitance retention when the current density increased to 3 A·g^−1^). Moreover, the HNT/C-1:1.5-800 electrode demonstrated excellent cycling stability with only 1.5% capacitance loss after 500 cycles. Furthermore, the HNT/C-1:1.5-800 exhibited high energy density of 12.5–20.28 Wh·Kg^−1^ and high power density of 0.1−1.5 kW·Kg^−1^. Hence, the preparation of carbon-based nanocomposites using this feasible strategy will provide vast opportunities for energy storage applications. Furthermore, the preparation of a renewable carbon precursor with a higher biomass content, the development of porous biobased materials into nanomaterials with different dimensions and porosity, and their application in functional applications, including supercapacitors, are of significance.

## Figures and Tables

**Table 1:** Porosity and electrochemical properties of the samples.

Sample	*S_BET_* m^2^·g^−1^	*V_total_* cm^3^·g^−1^	Pore Size nm	*C_KOH_* F·g^−1^
HNT/C-1:1.5-700	211	0.24	10.3	95
HNT/C-1:2.0-700	245	0.31	8.8	87
HNT/C-1:2.5-700	254	0.27	9.7	79
HNT/C-1:1.5-800	248	0.20	5.8	136
HNT/C-1:1.5-800 with PVDF and carbon black	186	0.14	7.1	136
HNT/C-1:1.5-900	156	0.06	42.8	107
Pristine C	122	-	-	68
Pristine HNT	115	0.27	13.7	50

*S_BET_*: specific surface area calculated by Brunauer–Emmett–Teller (BET) equation; *V_total_*: single point desorption total pore volume of pores; pore size: the pore size was determined from BJH adsorption average pore diameter; *C_KOH_*: specific capacitance obtained at current density of 1 A g^−1^ in 6 M KOH.

**Table 2:** Comparison of the electrochemical properties of different materials based on phenolic resin or HNTs.

Materials		Current Density	Specific Capacitance (F g^−1^)	Cycle Life	Capacitance Retention (%)	Reference
**Phenolic resin** **based materials**	AC	1 mA cm^−2^	160	-	-	[18]
	N-OMC	0.1 A g^−1^	216	10,000	>100%	[10]
	HPC	2 A g^−1^	215	10,000	98.2	[50]
**HNT** **based materials**	GQD-HNT	6 A g^−1^	258	5000	88	[51]
	HNT/rGO	0.1 A g^−1^	22.73	50	84.7	[25]
	H-PANI-PSS-PANI	0.5 A g^−1^	137	-	-	[24]
HNT/C		1 A g^−1^	136	500	98.5	This work

## Supplementary Materials

The following are available online at https://www.mdpi.com/2079-4991/10/9/1703/s1, Figure S1: XRD patterns of HNT/C, Figure S2: Rate performance of HNT/C-1:1.5-800 at current density of 0.2, 0.5, 1, 2 and 3 A·g^−1^, Figure S3: Self-discharge voltage of HNT/C-1:1.5-800 after constant current charging to 0.34 V, Figure S4: The electric double-layer capacitance diagram of HNT/C-1:1.5-Y based on the CV curve at 5 mV/s, Figure S5: The EIS fitting of HNT/C-1:1.5-900 and insets is the enlarged high-frequency region of the plots, Figure S6: The Ragone plots of the symmetric cell of HNT/C.
