# Supplementary material for: Preparation and Performance of Porous Carbon Nanocomposite from Renewable Phenolic Resin and Halloysite Nanotube
Source: Nanomaterials (Basel). 2020 Aug 29;10(9):1703. doi: 10.3390/nano10091703 (PMC7560184; doi:10.3390/nano10091703)
Supplement: Supplementary file 1 [file nanomaterials-10-01703-s001.pdf]

nanomaterials

Supplementary Materials

Preparation and Performance of Porous Carbon Nanocomposite from Renewable Phenolic Resin and Halloysite Nanotube

Xiaomeng Yang [1,†], Xiaorui Zeng [1,2,†], Guihong Han [1], Dong Sui [3], Xiangyu Song [1,*] and Yongsheng Zhang [1,*]

1 School of Chemical Engineering, Zhengzhou University, Zhengzhou 450001, China; yangxiaomeng@126.com (X.Y.); xrzeng@126.com (X.Z.); guihong-han@hotmail.com (G.H.)
2 Zhengzhou No. 9 High School, 21 Nongye Road, Zhengzhou 450002, China
3 Key Laboratory of Function-Oriented Porous Materials, College of Chemistry and Chemical Engineering, Luoyang Normal University, Luoyang 471934, China; suidonghy@mail.nankai.edu.cn
* Correspondence: xysong@zzu.edu.cn (X.S.); yzhang@zzu.edu.cn (Y.Z.)
† These authors contributed equally to this work.

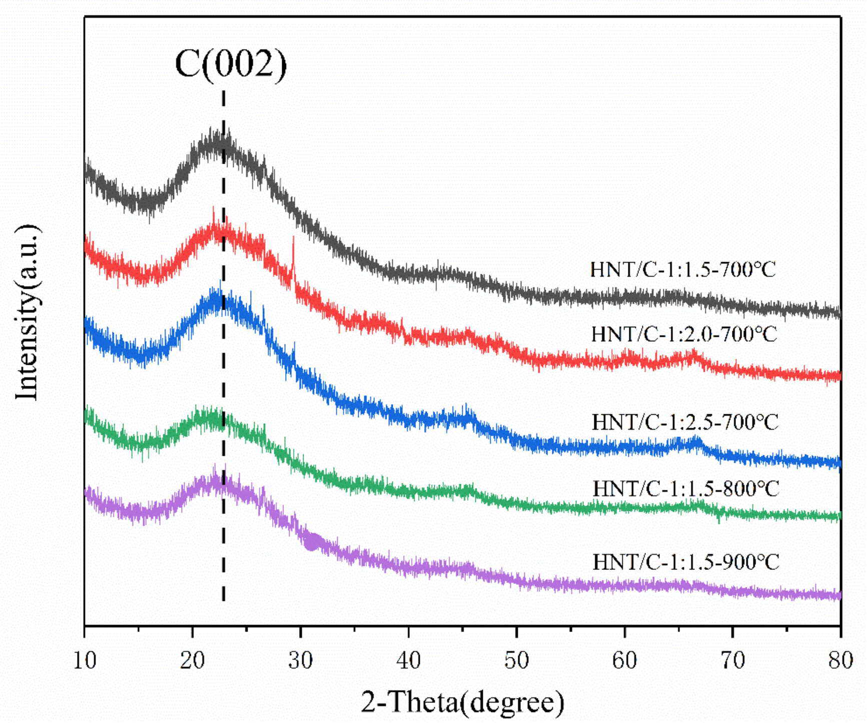

Figure S1. XRD patterns of HNT/C.

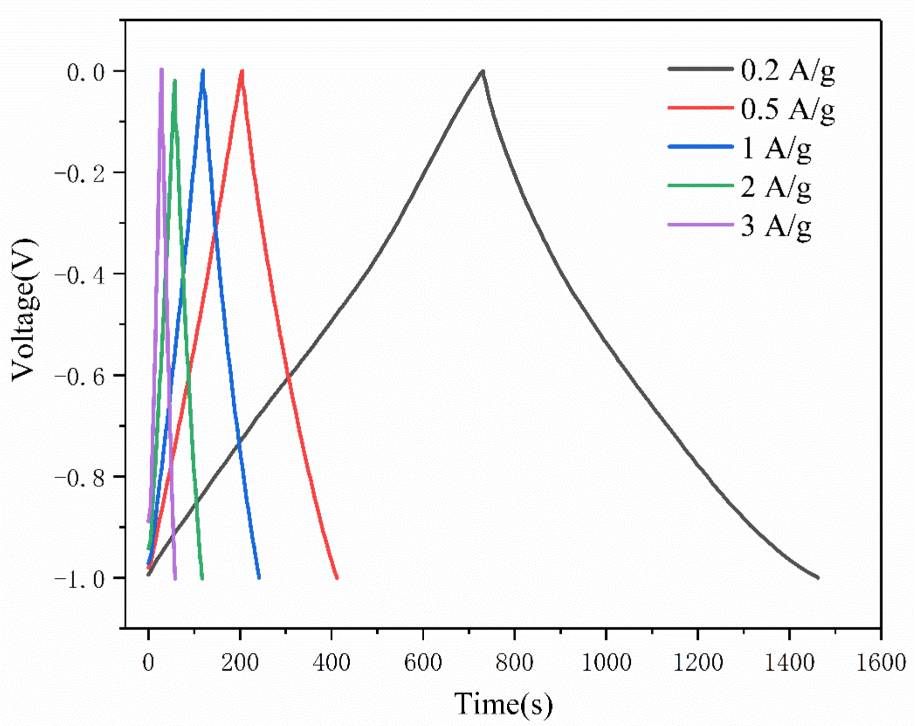

Figure S2. Rate performance of HNT/C-1:1.5-800 at current density of 0.2, 0.5, 1, 2 and 3 $A \cdot g^{-1}$.

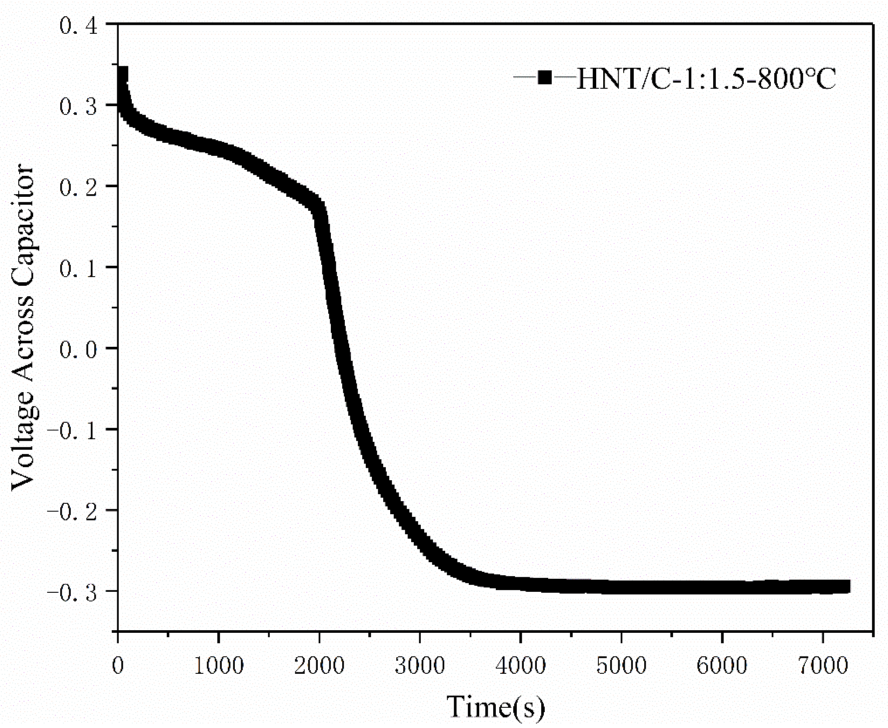

Figure S3. Self-discharge voltage of HNT/C-1:1.5-800 after constant current charging to 0.34 V.

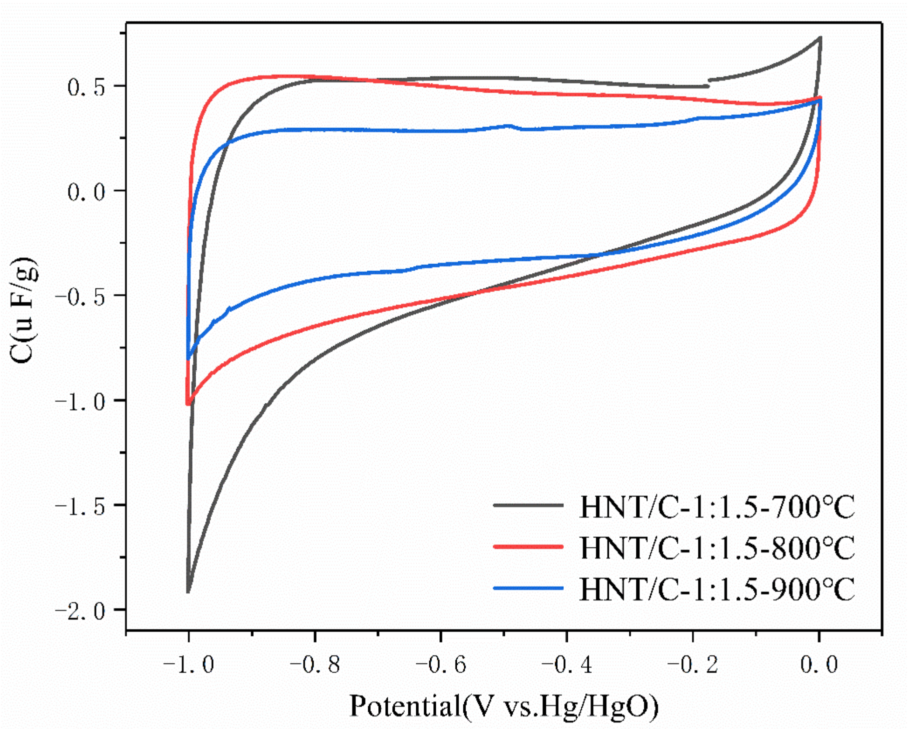

Figure S4. The electric double-layer capacitance diagram of HNT/C-1:1.5-Y based on the CV curve at 5 mV/s.

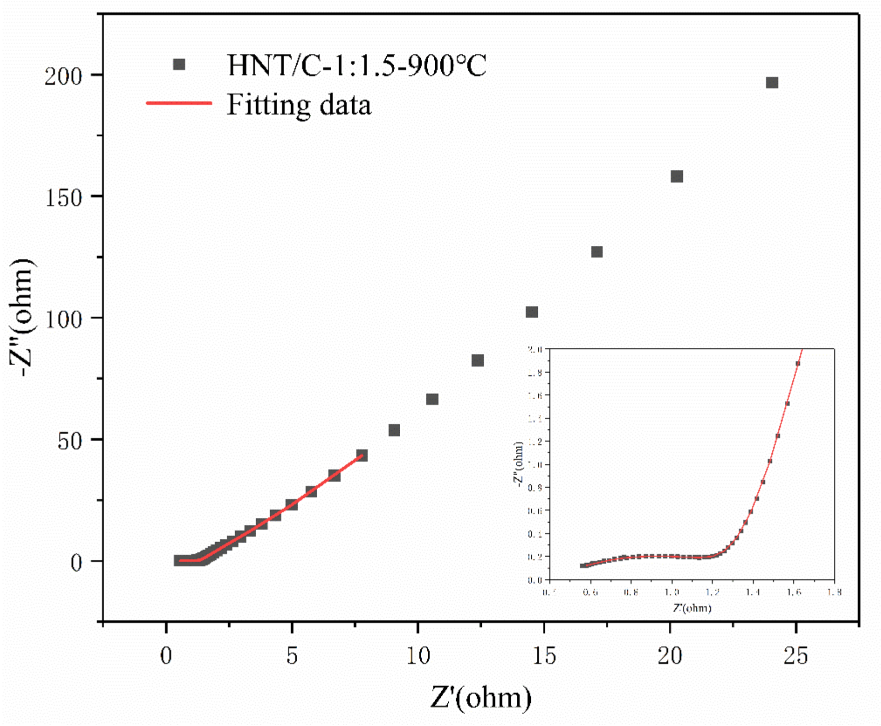

Figure S5. The EIS fitting of HNT/C-1:1.5-900 and insets is the enlarged high-frequency region of the plots.

Figure S6 shows the Ragone plots of the samples at different composite proportions and different carbonization temperatures. At the same power density, the energy density of the sample decreases with the increase of the composite ratio, which is consistent with the test results in the three-electrode system. The HNT/C-1:1.5-700 symmetric supercapacitor could supply maximal energy density of

16.97 Wh·Kg^{-1} where the power density was 100 W·Kg^{-1}. This value is higher than HNT/C-1:2.0-700 and HNT/C-1:2.5-700 of 15.89 Wh·Kg^{-1} and 14.45 Wh·Kg^{-1}, respectively. HNT/C-1:1.5-800 exhibit the maximum power density at different carbonization temperatures, 20.28 Wh·Kg^{-1}, which is higher than HNT/C-1:1.5-700 and HNT/C-1:1.5-900 of 16.97 Wh·Kg^{-1} and 17.14 Wh·Kg^{-1}, respectively. This obervation is speculated that the carbonization degree of the material is enhanced along with the rising temperature and forms more porous structure. However, as the temperature rises to 900 °C, the pore structure of the material collapses and large pores are generated. This result displays the lower electrochemical performance of HNT/C-1:1.5-900 compare to HNT/C-1:1.5-800. Therefore, HNT/C-1:1.5-800 demonstrates potential as an excellent electrode material for supercapacitance.

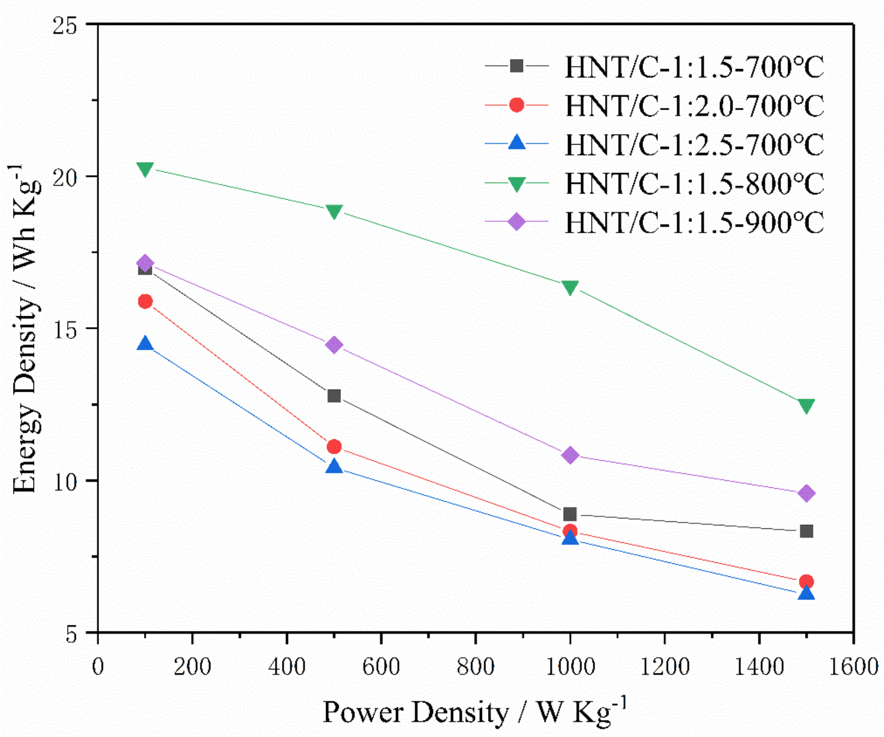

Figure 6. The Ragone plots of the symmetric cell of HNT/C.
